# Disturbed bone remodelling activity varies in different stages of experimental, gradually progressive apical periodontitis in rats

**DOI:** 10.1038/s41368-019-0058-x

**Published:** 2019-08-26

**Authors:** Ruoshi Xu, Daimo Guo, Xuedong Zhou, Jianxun Sun, Yachuan Zhou, Yi Fan, Xin Zhou, Mian Wan, Wei Du, Liwei Zheng

**Affiliations:** 1 State Key Laboratory of Oral Diseases, National Clinical Research Center for Oral Diseases, Department of Cariology and Endodontology, West China Hospital of Stomatology, Sichuan University, Chengdu, China; 2 State Key Laboratory of Oral Diseases, National Clinical Research Center for Oral Diseases, Department of Pediatrics, West China Hospital of Stomatology, Sichuan University, Chengdu, China

**Keywords:** Cell biology, Biological models

## Abstract

Bone remodelling keeps going through the lifespan of human by bone formation and bone resorption. In the craniofacial region, mandibles act as the main force for biting and chewing, and also become susceptible to a common bone-loss disease, namely, apical periodontitis, once infected dental pulp is not treated timely, during which bone resorption occurs from the apical foramen to the apical bone area. Although conventional root canal treatment (RCT) can remove the most of the infection, chronical apical periodontitis due to incomplete removal of dental pulp and subsequent microleakage will become refractory and more challenging, and this process has scarcely been specifically studied as a bone remodelling issue in rat models. Therefore, to study chronical and refractory apical periodontitis owing to incomplete cleaning of infected dental pulp and microleackage in vivo, we establish a modified rat model of gradually progressive apical periodontitis by sealing residual necrotic dental pulp and introducing limited saliva, which simulates gradually progressive apical periodontitis, as observed in the clinical treatment of chronical and refractory apical periodontitis. We show that bone-loss is inevitable and progressive in this case of apical periodontitis, which confirms again that complete and sound root canal treatment is crucial to halt the progression of chronical and refractory apical periodontitis and promote bone formation. Interestingly, bone remodelling was enhanced at the initial stage of apical periodontitis in this model while reduced with a high osteoblast number afterwards, as shown by the time course study of the modified model. Suggesting that the pathological apical microenvironment reserve its hard tissue formation ability to some degree but in a disturbed manner. Hopefully, our findings can provide insights for future bone regenerative treatment for apical periodontitis-associated bone loss.

## Introduction

During human adulthood, bone keeps remodelling by continuous bone formation and bone resorption.^1^ Precise coordination of ossification by osteoblasts and bone resorption by osteoclasts contributes to physiological bone maintenance.^2^^,3^ To maintain the integrity of bone, bone remodelling occurs through three well-organized phases during the bone remodelling cycle.^4^ Briefly, osteoclasts are initially formed to resorb bone, then reversely, osteoblasts are subsequently activated, and finally new bone is formed to make up for the bone loss, initially through well-balanced activity of osteoclasts and osteoblasts.^4^ Disturbed bone remodelling cycles or imbalanced activity of osteoclasts and osteoblasts are often associated with pathological bone disease, arthritis^5^ and osteolysis,^6^ for example.

Individuals are susceptible to tooth caries and tooth injuries during their lifespan. Once dental pulp is irreversibly involved in infection and necrosis, apical bone loss can occur if RCT is not effectively operated.^7–9^ The infected root canal system is classified into four grades: grade I (clean), grade II (non-infectious), grade III (infectious) and grade IV (severely infectious).^10^ Anatomy of the root canal system, infection grade and root canal preparation are the key factors of infection control.^10^ After multiple visits for state-of-the-art root canal preparation, a severely infectious root canal with a complicated anatomy of the root canal system can still be associated with chronic apical periodontitis featured by large apical lesions and root resorption due to impossibly complete removal of infected dental pulp, in which cases infection control by RCT cannot always be successful.^10^ Additionally, incomplete sealing of the root canal system from microleakage or the introduction of irritants from the oral cavity after RCT aggravates chronic apical periodontitis.^11^ Uncontrolled chronic apical periodontitis then becomes progressively refractory, and then endodontic microsurgery or tooth extraction^12^ might be the subsequent optional treatment.^13,14^ According to the AAE (American Association of Endodontists), the United States alone has more than 14 million cases of RCT per year; however, not all RCTs are successful.^12,15,16^ Based on strict criteria in systematic reviews of the literature, the rate of failed primary RCT was reported to be 15%–32%,^17^ and that of failed secondary RCT was 23%,^18^ mainly due to re-infection and tooth fracture.^19^ Along with infection control based on strict criteria, improving the apical bone microenvironment, inducing apical bone regeneration and, ultimately, treating chronic and refractory apical periodontitis and thereby saving the tooth is expected in endodontic microsurgery but challenging in current nonsurgical treatment for chronic apical periodontitis.^14^ Additionally, chronic apical periodontitis is widely studied as an infectious disease but seldom discussed as an issue of bone remodelling. Therefore, our study focused on bone remodelling in the context of chronic and refractory apical periodontitis due to uncleaned infected pulp and microleakage in order to understand how bone defects occur and proceed in the presence of limited persistent irritants. This study hopefully will provide insights for biological approaches during nonsurgical endodontic treatment to additionally halt apical bone-loss progression and further increase the chances of tooth preservation.^8^

Multi-microbial infection from dental pulp is the main cause of apical periodontitis.^20^ Infiltration of inflammatory cells,^21^ monocytes^22,23^ and macrophages,^24^ for example, and production of cytokines^25^ are reported to induce apical periodontitis. As apical periodontitis proceeds, bone destruction is the prominent feature in diagnosing apical periodontitis and assessing prognosis after treatment, but has only been partially studied as an issue of bone remodelling specifically in the context of chronic and refractory apical periodontitis.^26^ To study bone lesions in general apical periodontitis, rat, dog^27^ and mouse^28^ models have been previously established to meet the needs of various studies. Pulp exposure^29^ to the oral environment of rats causes apical periodontitis in a direct way, which was mainly applied to study inflammation^30,31^ or immune reactions.^32^ Modifications were reported as the inoculation of mixed bacteria directly into exposed pulp^33,34^ or plaque contamination,^28^ leading to severe apical bone lesions. A rat model to simulate the progression of chronic and refractory apical periodontitis due to incomplete RCT or microleakage has rarely been reported. Additionally, time course studies of bone remodelling activity once apical periodontitis occurs are scarcely reported in detail.

Our research aimed to develop a modified rat model to specifically study chronic and refractory apical periodontitis caused by incomplete root canal treatment from the perspective of clinical practice and hypothesized that apical bone is reduced increasingly more severely and remodelling is gradually decreased as apical periodontitis progresses. By replaying the same endodontic procedures as performed in daily clinical practice, we generated a chronic-and-refractory-apical-periodontitis-like bone lesion induced by residual dental pulp and microleakage. Similar pathological features of chronic and refractory apical periodontitis can be convincingly observed in this model, such as a widened periodontal ligament, a gradually progressive bone lesion, periradicular granuloma, root resorption and bone-like cementum hyperplasia. Based on strict criteria, we describe this modified model as gradually progressive apical periodontitis characterized by induced chronic-and-refractory-apical-periodontitis-like bone lesions. In conclusion, we first established a disease model in rats with gradually progressive apical periodontitis that simulates the progression of chronic and refractory apical periodontitis. During a time course study from the model, we found that bone remodelling is transiently enhanced and subsequently reduced with a high osteoblast number and that the hard tissue formation ability of such a pathological apical microenvironment is reserved to some degree in a disturbed manner.

## Results

### Human chronic apical periodontitis is associated with disturbed bone remodelling

**Fig. 1:**
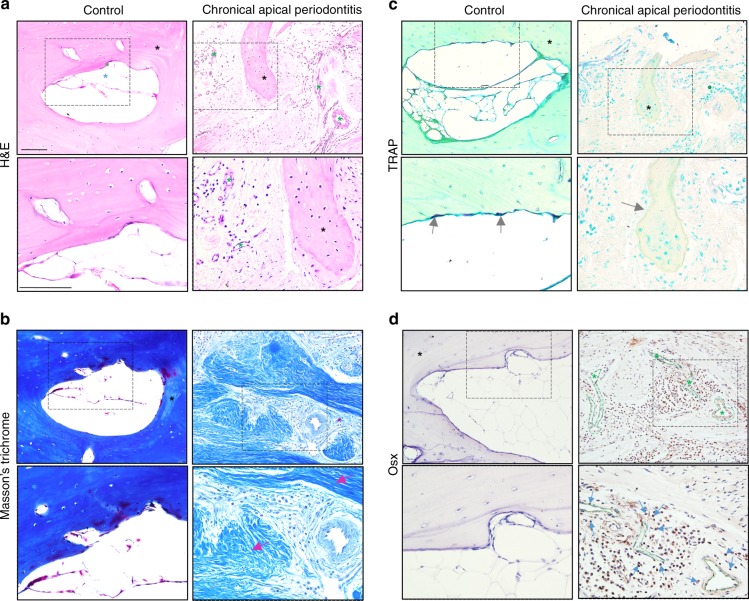
Bone remodelling is disturbed in the periapical region of human apical periodontitis. **a**–**d** Representative images of histological and immunohistochemistry staining with the indicated antibodies on serial sections of human apical region tissue from patients without (control) or with chronic apical periodontitis. **a** H&E staining shows lining cells (grey asterisk), adipose tissue (blue asterisk) in the control and increased blood vessels and decreased bone tissue in apical periodontitis tissue. **b** Masson’s trichrome staining shows increased collagen fibres (pink arrow). **c** TRAP staining shows decreased osteoclasts (grey arrow). **d** Immunohistochemical staining of Osx shows increased osteoblasts (blue arrow) surrounding the blood vessels (green dotted lined area). The magnified framed region is shown below. Bone tissue (black asterisk), blood vessels (green asterisk); scale bar: 100 µm

To study chronic and refractory apical periodontitis, a pathological analysis by histological staining of this kind of apical lesion was first performed. As is known, chronic and refractory apical periodontitis with a complicated root canal system is hardly fixed by simple RCT due to an inaccessible infected dental pulp. Therefore, the uncontrolled apical lesions from the above-referenced patients after RCT were then removed by microsurgery involving periradicular curettage, during which apical lesions were harvested and processed after the patients agreed. Normal apical bone tissue was harvested from patients who required a bone repair procedure to remove the sharp apical bone crest between roots after extraction of the third molar without apical periodontitis. By H&E and Masson’s trichrome staining, bone tissue and bone marrow were scarcely observed in the apical areas of chronic and refractory apical periodontitis but were largely replaced by soft tissue characterized by inflammation-related cells, angiogenesis and dense collagen fibres (Fig. 1a, b). Apical periodontitis is largely a bone-loss disease^35^; therefore, it is hypothesized that bone remodelling activity is inhibited in patients with apical periodontitis. To test this hypothesis, osteoblasts and osteoclasts are visualized by staining. TRAP-positive osteoclasts were not detected at the apical bone surface, while Osterix (Osx)-positive cells, markers for osteoblasts, were largely increased and associated with increased blood vessels in the apical bone-loss area (Fig. 1c, d). Taken together, chronic and refractory apical periodontitis lesions with long-lasting bone radiolucent X-ray images after RCT are associated with reduced bone remodelling and filled with an increased osteoblast number and volume of collagen fibres, which guided us to form the following hypothesis: Chronic and refractory apical periodontitis lesions are characterized by reduced bone remodelling activity alone and may become more severe once bone loss initiates.

### Modified experimental apical periodontitis largely simulates clinical chronic apical periodontitis

**Fig. 2:**
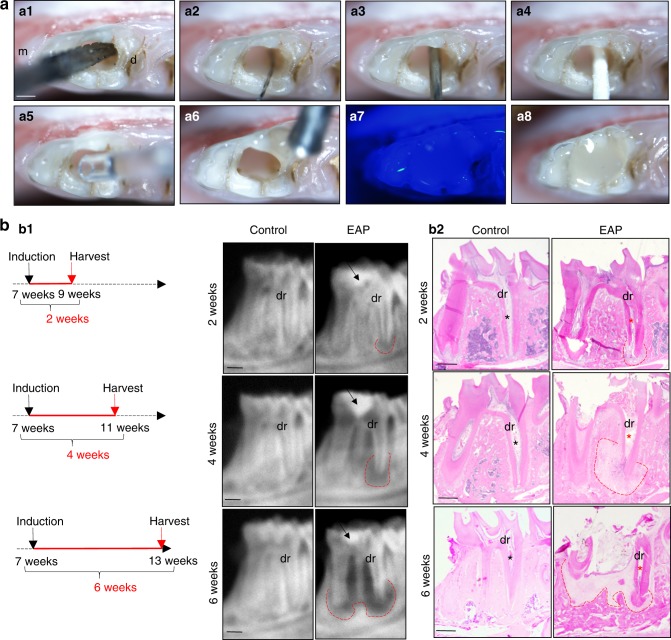
Modified EAP in rats leads to progressive bone loss. **a** Oral images of modified endodontic procedures of EAP to the mandibular first molar. **a1** Access to the pulp chamber gained through the distal root canal. **a2** Pulp of the distal root canal largely removed. **a3** Irrigation by PBS. **a4** Drying by paper points. **a5** Addition of PBS to the distal root canal. **a6**–**8** Sealing of the cavity. Scale bar: 500 µm. **b** Time course of the observation. **b1** Schematics of the time course of induction and harvest. EAP was induced in seven-week-old rats. X-ray images after 2 weeks, 4 weeks and 6 weeks of observation are shown on the right side. **b2** HE staining of the mandibular first molar; residual distal dental pulp (red asterisk) was sealed as persistent mild irritants by composite resin (**b1** black arrow) compared to the normal pulp (black asterisk). Red dotted area, radiolucent apical periodontitis lesion in **b1**–**2**. m, mesial; d, distal; dr, distal root of the mandibular first molar. Scale bar: 100 µm

To further understand persistent bone loss in chronic and refractory apical periodontitis lesions with indications for periradicular curettage, a modified apical periodontitis animal model was established to simulate the progression of human chronic and refractory apical periodontitis in vivo. With a combination of previously described apical periodontitis animal models^27,28,36^ and modification according to the purpose of our research, we generated gradually progressive apical periodontitis bone lesions with persistent but mild irritants from the root canal due to uncleaned dental pulp and the introduction of saliva (Fig. 2a and Supplementary Fig. 2b). Briefly, the distal dental pulp was exposed and largely removed without physically destroying the rest of the dental pulp in the mesial chamber and other root canals. Then, the distal root canal was irrigated with PBS to remove residual floating pulp, the bleeding was stopped by drying, followed by moistening with PBS to prevent the apical bone tissue from drying. After introducing saliva, the attached distal pulp residual was sealed and largely segregated from infection of the oral cavity, therefore keeping the necrotic pulp residual and saliva as a persistent and mild irritant to apical bone tissue in order to recapitulate the progression of apical periodontitis due to uncleaned dental pulp and microleakage. Since timely RCT is associated with better bone volume reservation, we modified the hypothesis that apical bone responds differently to irritants at various stages during the progression of chronic and refractory apical periodontitis. To test the hypothesis and study the progression of chronic and refractory apical periodontitis in rats, we set three time points of observation after the induction of experimental apical periodontitis (EAP) to analyse apical bone tissue (Fig. 2b1). To simulate a clinical examination, rough images of X-ray films of distal root were taken to show that an apical radiolucent lesion surrounded the distal root (Fig. 2b1), owing to residual necrotic pulp and sealed saliva (Figs. 2b2 and 5d), which looks similar to the clinical observation corresponding to variously severe cases of chronic apical periodontitis with complicated root canal systems due to uncleaned dental pulp and microleakage.^11^

### Modified EAP leads to gradually progressive bone loss

**Fig. 3:**
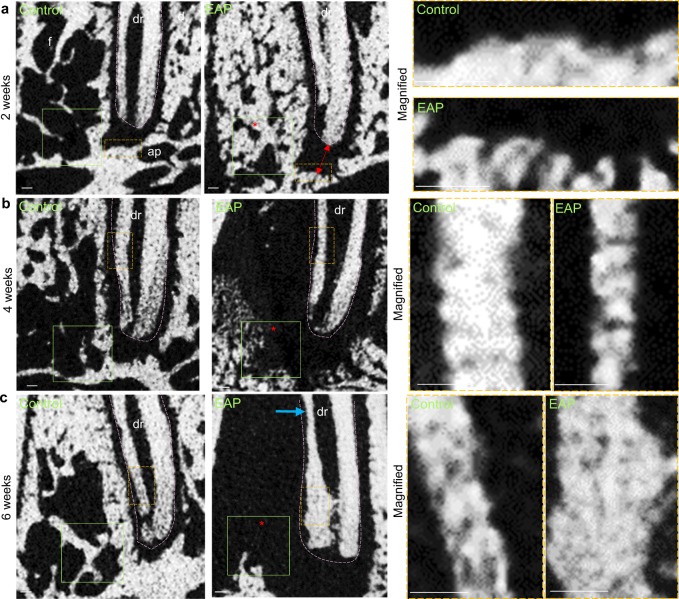
Modified EAP in rats leads to progressive bone loss. Representative µCT images of the distal apical region of the mandibular first molar of rats in two dimensions with or without induction of EAP under three time courses of observation. **a** Two weeks after induction, the discontinuous surface of the lamina dura (yellow frame), widened PDL (double arrow) and increased bone density (asterisk) are indicated. **b** Four weeks after induction, bone-loss lesion (asterisk) and slightly reduced root canal wall (yellow frame) are indicated. **c** Six weeks after induction, bone-loss lesion (asterisk), reduced wall thickness (blue arrow) and thickened root canal wall (yellow frame). m, mesial; d, distal; c, crown; mr, mesial root; dr, distal root (pink dotted line); f, furcation area; ap, apical bone; av, alveolar bone. Green boxes (**a**–**c**) are ROIs for bone parameter analysis (Fig. 4). Scale bar: 100 µm

**Fig. 4:**
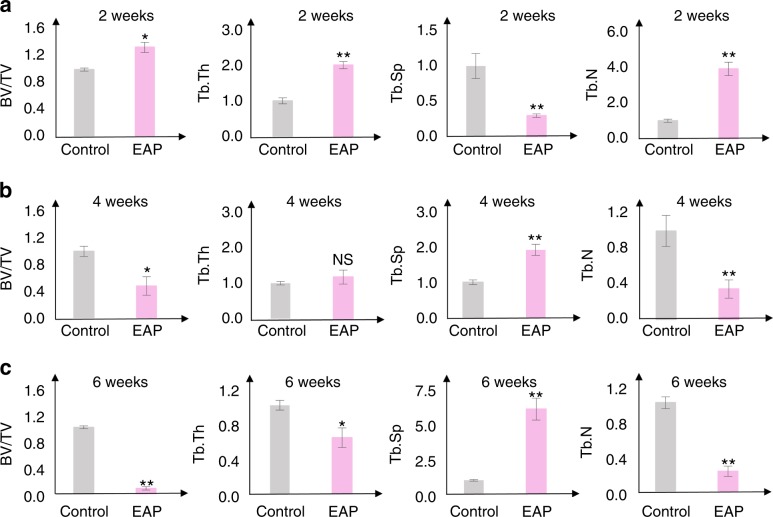
Bone parameter analysis of µCT of EAP in rats. µCT analysis of BV/TV, Tb.Th, Tb.N and Tb.Sp of the ROI corresponding to Fig. 3, **a** 2 weeks after induction, **b** 4 weeks after induction and **c** 6 weeks after induction. ROIs are designated by green boxes (Fig. 3**a**–**c**) for the bone parameter analysis. Two-tailed Student’s *t*-test was used for comparisons between two groups. *P*-values less than 0.05 were considered significant with single asterisk, and *P* values less than 0.01 were considered significant with double asterisks

**Fig. 5:**
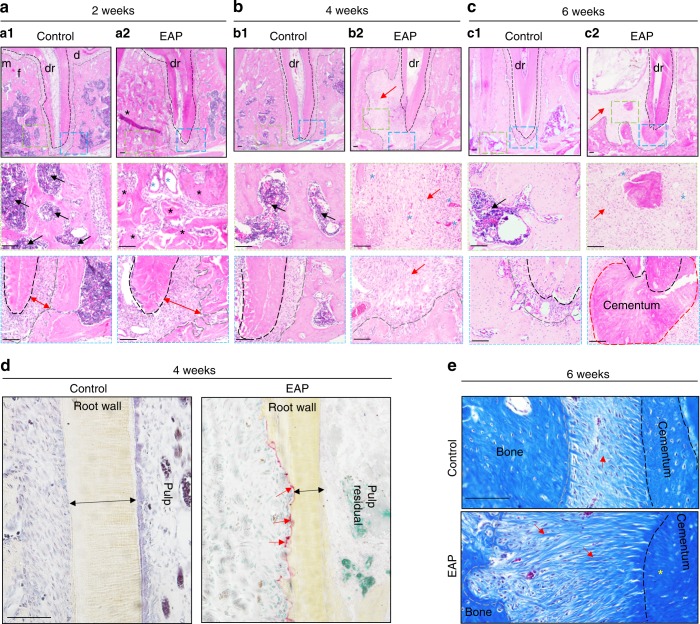
EAP in rats causes loss of bone structures and disorganized roots. **a**–**c** Representative H&E staining of the distal apical region of rats’ mandibular first molar with or without the induction of EAP. **a** Two weeks after induction, widened PDL (double arrow) and increased bone (black asterisk) are indicated. **b** Four weeks after induction, bone loss (red arrow) was found around the distal root. **c** Six weeks after induction, dramatic bone loss occupied the mesial and distal apical area (red arrow), thin root canal wall (white asterisk) and increased bone-like cementum tissue (red dotted line). Bone marrow (black arrow) was decreased, and blood vessels were increased (blue asterisk). **d** Representative TRAP staining on the root wall 4 weeks after induction. The root wall was thinner (double arrow), with resorption pits (red arrow). **e** Representative Masson trichrome staining of the apical area 6 weeks after induction. The collagen fibres were increased and thickened (red arrow). The cementum area was enlarged (yellow asterisk). Bone surface, grey dotted line; m, mesial; d, distal; dr, distal root (black dotted line); f, furcation area. Green/blue dotted boxes are shown at higher magnification below. Scale bar: 100 µm

Generally, the bone-loss area is progressively enlarged as the duration of irritants becomes longer, while the bone density varies in different stages as shown by µCT analysis (Figs. 3 and 4). At the initial stage, 2 weeks after induction, the apical area showed mild bone-loss lesions characterized by increased width of the periodontal ligament (PDL) beside the apex and an uneven surface of compact bone or lamina dura (Fig. 3a). The apical bone area adjacent to the widened apical periodontal ligament showed increased bone density, which was characterized by an increased ratio of bone volume to total volume (BV/TV), increased trabecular thickness (Tb.Th), increased trabecular number (Tb.N), and decreased trabecular spacing (Tb.Sp) (Fig. 4a). At the middle stage, 4 weeks after induction, the bone-loss area expanded and destroyed most of the alveolar bone of the distal root, as shown by a large cavity around the distal root, which was further analysed as decreased BV/TV, decreased Tb.Th, decreased Tb.N, and increased Tb.Sp of the apical area (Figs. 3b and 4b). Additionally, the distal root showed a slightly decreased thickness of the root canal wall, suggesting that root resorption occurred (Fig. 3b). At the late stage, 6 weeks after induction, apical bone tissue was almost undetectable, which was characterized as decreased BV/TV, decreased Tb.Th, decreased Tb.N and increased Tb.Sp of the distal apical area (Figs. 3c and 4c). Moreover, the distal root showed a thin root canal wall at the middle part of the root and a thickened root canal wall at the apical part of the root, suggesting that root resorption occurred and additional hard tissue formed (Figs. 3c and 5c). Our data confirm what has been observed during clinical practice by using a rat model in which timely and sound root canal treatment is crucially required for apical periodontitis patients, since apical periodontitis in EAP cannot be self-repaired and apical periodontitis with remaining necrotic dental pulp and saliva leads to further apical bone loss and root resorption. Interestingly, the apical bone density increases shortly after induction but is followed by progressive bone loss when the apical area is persistently exposed to mild irritants.

### EAP causes loss of apical area structures and a disorganized distal root

To understand the detailed bone morphology of experimental apical periodontitis, a histological analysis was applied to the apical area with or without induction. Two weeks after induction, the apical periodontal ligament became wider, the integrity of the compact bone adjacent to the periodontal ligament, or the lamina dura, was broken or became uneven, and part of the bone marrow was replaced by bone tissue, blood vessels and fibres (Fig. 5a). Four weeks after induction, a dramatically large bone-loss lesion destroyed most of the distal apical bone and partial furcation area. Bone marrow could be scarcely detected in the involved alveolar bone, and fibres and blood vessels were filled in place of bone tissue (Fig. 5b). Root walls were initially found by TRAP staining to be thinner than the control at this stage (Fig. 5d). Six weeks after induction, tooth fracture frequently occurred, the floor of the pulp chamber broke, the root wall became even thinner, a thickened cementum with dense collagen perforating the fibre at the apical foramen formed, the gingiva receded to the middle third of the root, the periodontal attachment was lost, and the alveolar bone was resorbed from the rim to the floor and from the apical area of the distal root to that of the mesial root (Fig. 5c, e). Taken together, our data successfully simulated the histological analysis of clinical cases of chronic and refractory apical periodontitis by reproducing clinical features in a rat model that started from a widened apical PDL, then proceeded to gradual loss of bone structures, and finally progressed to disorganized roots and increased collagen fibres in the modified model.

### EAP initially promotes but subsequently reduces bone remodelling

**Fig. 6:**
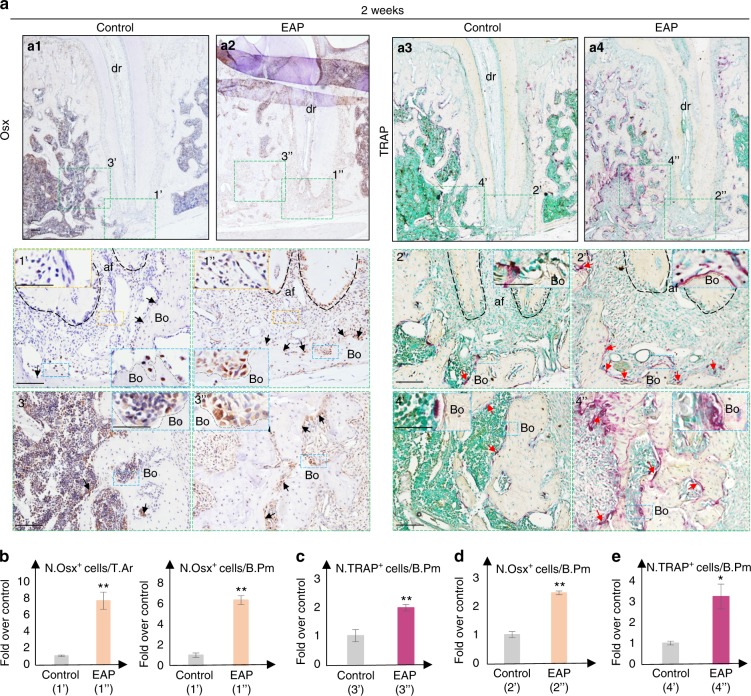
EAP initially promotes bone remodelling. **a** Representative image of immunohistochemistry staining with the indicated antibody and TRAP staining on serial sections of the rats’ distal root apical region two weeks after induction of EAP. **a1**–**a4** Area of distal apical bone: green dotted boxes are magnified below, and the ROIs for statistical analysis (**b**–**e**). **1’**, **1”**, **b** Increased Osx-positive cells in the apical ligament (yellow frame) and on the apical bone surface (black arrow, blue frame). **2’**, **2”**, **c** Slightly increased TRAP-positive cells on the apical bone surface (red arrow, blue frame). **3’**, **3”**, **d** Increased Osx-positive cells on the bone surface of the adjacent area to the apical foramen (black arrow, blue frame). **4’**, **4”**, **e** Increased TRAP-positive cells on the bone surface of the area adjacent to the apical foramen (red arrow, blue frame). Scale bar: 100 µm. Yellow/blue boxes are presented with a scale bar of 50 µm. Bo, bone; dr, distal root (black dotted); a, apical foramen, bone surface (grey dotted line)

**Fig. 7:**
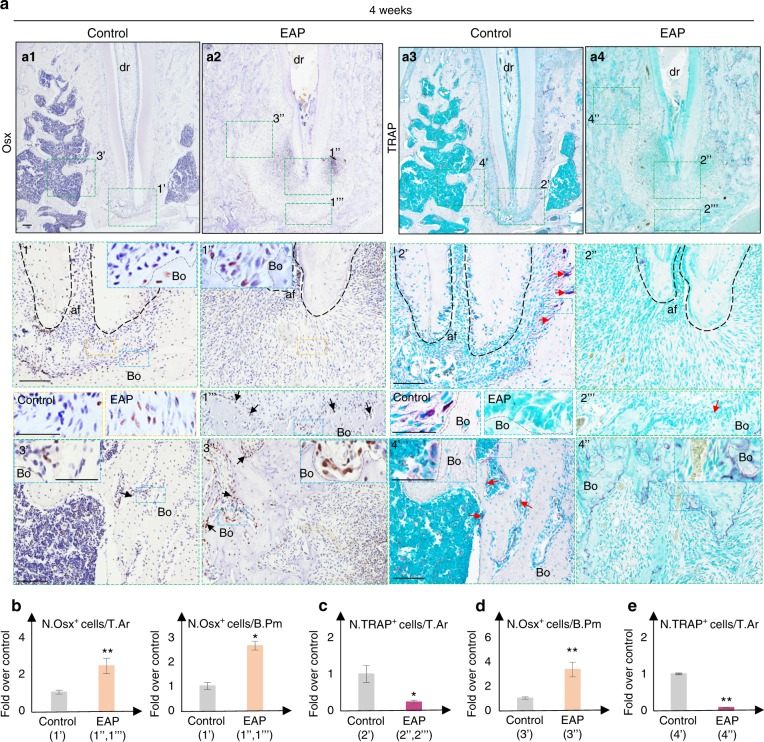
EAP reduces bone remodelling at the latter stage. **a** Representative images of immunohistochemistry staining of Osx and TRAP staining on serial sections of the rats’ distal root apical region 4 weeks after the induction of EAP. **a1**–**a4** Green dotted boxes are magnified below, and the ROIs for statistical analysis (**b**–**e**). **1’**, **1”**, **b** Increased Osx-positive cells in the apical ligament area (yellow frame) and on the apical bone surface (black arrow, blue frame). **2’**, **2”**, **c** Decreased TRAP-positive cells on the apical bone surface (red arrow, blue frame). **3’**, **3”**, **d** Increased Osx-positive cells on the bone surface of the area adjacent to the apical foramen (black arrow, blue frame). **4’**, **4”**, **e** Decreased TRAP-positive cells adjacent to the apical foramen (red arrow, blue frame). Scale bar: 100 µm. Yellow/blue boxes are presented with a scale bar of 50 µm. Bo, bone; dr, distal root (black dotted area); af, apical foramen, bone surface (grey dotted line)

Uncontrollable bone-loss progression shown in EAP suggests that bone remodelling is disturbed. Increased bone density at the initial stage and decreased bone volume at later stages suggests that bone remodelling in response to irritants varies at different stages. Since tooth fracture occurred frequently 6 weeks after induction and the distal apical lesion was interfered with by subsequent irritants from other roots and periodontitis, analysis of bone remodelling was performed in the first two stages. To determine how bone remodelling is disturbed in terms of osteoblasts and osteoclasts, visualization of osteoblasts and osteoclasts in vivo was obtained by local immunohistochemistry staining (Figs. 6a and 7a) and quantification (Figs. 6b–e and 7b–e) of osteoblasts and osteoclasts at the apical bone surface and adjacent bone marrow.

Generally, increased Osx-positive osteoblasts (Fig. 6a1’-1”,3’-3”,b, d) were associated with increased TRAP-positive osteoclasts 2 weeks after induction of apical periodontitis (Fig. 6a2’-2”,4’-4”,c, e), while increased Osx-positive osteoblasts (Fig. 7a1’-1”,3’-3”,b, d) were associated with slightly decreased TRAP-positive osteoclasts (Fig. 7a2’-2”,4’-4”,c, e) 4 weeks after induction, consistent with the human sample study in Fig. 1 and previous studies.^37,38^ By analysing the location of osteoblasts at the lamina dura, Osx-positive cells were mostly located along the surface of the lamina dura during homeostasis (Figs. 6a1’ and 7a1’). After induction of experimental apical periodontitis, increased Osx-positive cells were not only located on the bone surface but also located within the broadened periodontal ligament area 2 weeks after induction (Fig. 6a1”, b) or within the apical periodontitis lesion 4 weeks after induction (Fig. 7a1”-1”’, b), which failed to form new bone at the bone resorption pit. Conversely, within neighbouring bone marrow adjacent to the apical foramen, some of the Osx-positive cells were dispersed in the bone marrow, and the rest were located at the bone surface during homeostasis (Figs. 6a3’ and 7a3’). After the induction of experimental apical periodontitis, most of the increased Osx-positive osteoblasts were located along the bone surface, which were associated with a narrowed bone marrow space (Figs. 6a3” and 7a3”).

In sum, EAP showed initially enhanced bone remodelling activity with transient increased bone density and the activation of both osteoblasts and osteoclasts, which was followed by reduced bone remodelling with gradually progressive bone loss and a high number of osteoblasts in the middle stage (Supplementary Fig. 2c). The result of the middle stage was analogous to what is observed in chronic and refractory apical periodontitis patients, suggesting that a rat model with a similar bone remodelling status to that of patients was largely successfully established.

## Discussion

If root canal treatment is not received in time and there is a complicated root canal system, chronic apical periodontitis is more likely to become refractory owing to incomplete RCT and subsequently uncontrolled microleakage. Our study aimed to simulate chronic and refractory apical periodontitis due to uncleaned pulp residual and saliva and to investigate bone remodelling activity during gradually progressive bone loss in vivo, expecting to provide evidence and insights for future studies of further mechanisms and possible biological treatments of chronic and refractory apical periodontitis.

To target chronic and refractory apical periodontitis specifically, samples were harvested exactly from the above-described type of patients. By analysis of patients’ apical tissue obtained from the surgical treatment of chronic apical periodontitis, a brief histological blueprint of chronic apical periodontitis was obtained, and a simulated rat model was required for further study, which encouraged us to set up the model to achieve our purpose.

Compared to previous rat models, our modified model is identified by several characteristics that specifically meet the purpose of this study. Previous rat models were induced by pulp exposure with a dental round bur only, and the whole vital pulp in the root canal was left inside to study inflammation or immune reactions during apical periodontitis.^31,37,39–42^ To simulate refractory cases and rule out the impact from the complete vital dental pulp to apical areas, we utilized clinical endodontic procedures to largely remove the distal dental pulp. The pulp residual sealed by composite resin imitated the inaccessible pulp infection during root canal preparation in clinical cases with a complicated root canal system. Previous studies have reported that the bilateral mandibular first molar was exposed or the maxillary molar was extracted.^31,43^ Comparatively, to maximize the simulation of chronic and refractory apical periodontitis and minimize chewing pain in our study, maxillary molars were kept to largely maintain natural chewing as demonstrated by patients, and only right mandibular first molars were induced to keep the left side healthy for chewing and food intake. Moreover, as previously reported, four root canals of the mandibular first molar were performed, nickel-titanium rotary files were used to prepare the root canals, and they were finally filled with gutta-percha points using the single point method, the purpose of which was to set up a model of root canal treatment and to observe the prognosis of apical periodontitis,^43^ which is less applicable to our purpose. To rule out the interference of other roots of the first molar, we focused on the distal root and distal apical area as a single subject to study. For the rest of the roots, the vital pulp was kept, and the initial infection was limited to the orifice during induction; mesial apical periodontitis also occurred gradually and interfered with distal lesions in the late stage, which is one of the reasons that detailed analyses have been focused on the early and middle stages. Additionally, the distal root canal provides a better view and space for replicable endodontic procedures. Furthermore, early studies opened the pulp chamber and filed the distal root canal with #25 files to initiate apical periodontitis and study immune cells.^23^ To reserve the dental pulp residual as a mild irritant that simulates the inaccessible infected pulp after root canal treatment in cases with a complicated root canal system, by filing the distal root canal using more flexible #10-#15K files, irrigating, drying and sealing the pulp chamber by composite resin (Supplementary Fig. 2b), most of the distal dental pulp was removed, and residual necrotic dental pulp could be observed in histological images (Figs. 2b2 and 5d), contacting the apical area, which is commonly found in patients’ root canal systems after failed RCT.^11^ In our case, to utilize a rat model to specifically achieve the aim of this study to understand chronic and refractory apical periodontitis, we set up a modified rat model, which gave us an opportunity to document gradually progressive apical periodontitis during observation.

To study the bone remodelling status during chronic apical periodontitis, the analysis of gradually progressive apical periodontitis, as we established in the rat model, was documented. Several histological features were found to be similar to the human sample analysis at the beginning, with respect to clinical observations and other relative studies of apical periodontitis due to incomplete RCT, suggesting a largely successful simulation of clinical chronic and refractory apical periodontitis due to incomplete RCT. Initially, a widened periodontal ligament occurred two weeks after induction, which is similar to the early alarm of apical periodontitis we observed in X-rays from clinical practice. Subsequently, the histological features included a gradual bone-loss lesion, thin root wall and thickened cementum formation, which are similar to the radiological observations from radiolucent X-ray films and the surgical findings of root resorption and cementum hyperplasia in chronic apical periodontitis. Periradicular granulomas with small capillaries, fibroblasts and inflammatory cells shown 4 or 6 weeks after induction (Fig. 5b, c) were previously reported as 59.3% chronic apical periodontitis in a study of failed root canal treatment.^44^ By comparison, histological analysis and bone remodelling analysis at the middle stage of rats with apical periodontitis is consistent with human samples. Above all, this disease model of gradually progressive apical periodontitis is probably convincing for the subsequently study of chronic apical periodontitis due to the largely successful simulation of clinical features observed in chronic and refractory apical periodontitis due to incomplete RCT.

Based on the results of the rat model, the human samples from the surgical treatment of chronic apical periodontitis turned out to be in the middle or late stages of the rat study. To our surprise, before reduced bone remodelling (as found in human samples) occurred, an initially enhanced bone remodelling stage with transient increased bone volume was found in our modified rat model at an early stage. At the cellular level, it is suggested that apical bone tissue may have certain potential to self-repair bone loss at the initial stage of apical periodontitis by highly stimulated osteoblasts and osteoclasts during bone remodelling activity. Our data are consistent with clinical findings that timely RCT for apical periodontitis in the early stage of progression has a better prognosis than RCT performed at a severely late stage. Our result is also consistent with the tendency for the case number of root-end surgery to be decreased due to better clinical application of the shaping and cleaning of root canals in time.^35^ Mechanically, bone remodelling activity is a complicated event for maintaining bone volume, during which TGF-β plays a key role in orchestrating this event.^45^ By analysing the expression of pSmad2/3 in the apical area, TGF-β signalling was found to be aberrantly activated in chronic apical periodontitis from patients, which suggests that TGF-β signalling is involved (Supplementary Fig. 1a). Consistently, aberrant activation of TGF-β signalling was also observed at the initial stage in the rat model (Supplementary Fig. 1b). Interestingly, in contrast to human tissue, the initial stage in rats is characterized by increased pSmad2/3 cells located at the bone surface and within blood vessels in the apical area. Most likely, activated osteoclasts at the initial stage promote the release of TGF-β from the bone matrix and subsequently recruit mesenchymal stem cells to bone resorption pits to differentiate into osteoblasts and form new bone, which is consistent with the transiently increased bone volume and osteoblasts at this stage.^45^ Further study of TGF-β signalling is required to obtain a deeper understanding of chronic apical periodontitis and to provide insights for possible biological bone regeneration for bone loss in chronic apical periodontitis. At the late stage of the rat model, apical bone tissue showed uncontrolled and expanded bone loss, and bone-like cementum tissue with dense collagen perforating fibres was located at the apical foramen, extending from the cervical cementum and forming at the outer surface of the dental root (Fig. 5e), which is advantageous for premature roots with large apical foramens. The main tasks to treat chronic and refractory apical periodontitis are infection control and bone lesion fixation. Infection control depends on RCT, while bone lesion fixation in nonsurgical treatment relies on hosts themselves. Our study of bone remodelling focuses on the latter issue. We found that bone formation is transiently enhanced at the initial stage of apical periodontitis and that cementum formation is enhanced at the late stage of apical periodontitis even if root resorption happens, suggesting that the pathological apical microenvironment reserve hard tissue formation ability to some degree but in a disturbed manner. The unknown pathological apical microenvironment deserves further study to reveal how bone remodelling is regulated and to discover a promising biological approach for nonsurgical treatment for bone lesion fixation in chronic and refractory apical periodontitis.

## Materials and methods

### Ethics statement

The research follows guidance issued by the Institutional Animal Care and Use Committee at Sichuan University. The study was approved by the ethical committees of West China School of Stomatology, Sichuan University and State Key Laboratory of Oral Diseases. Human apical tissues were harvested with an informed consent procedure from West China Hospital of Stomatology. The apical periodontitis tissue was harvested during periradicular curettage microsurgery from patients who had persistent radiolucent images after RCT and received subsequent microsurgery of periradicular curettage to further remove apical lesions. Normal apical bone tissue was harvested from patients who required a bone repair procedure to remove the sharp apical bone crest between roots after extraction of the third molar without apical periodontitis. SPFSD adult rats at 7 weeks were purchased from Dashuo Laboratory Animal Company (Sichuan, China). To rule out the effect of oestrogen on bone volume in female rats, male rats were selected as subjects in this research. All rats were fed and sacrificed with care.

### Endodontic procedures in the induction of experimental apical periodontitis

Preparations were performed before the induction of experimental apical periodontitis, and the working length of the distal root canal was measured in the extracted mandibular first molar from Sprague Dawley rats at 7 weeks old, with an average of 2–3 mm, consistent with a previous study.^43^ The #10-#15K file was soft enough to reach the whole working length and remove as much as dental pulp as possible by filing up and down. Sprague Dawley rats at 7 weeks old were anaesthetized by a careful intraperitoneal injection using 10% chloral hydrate. We accessed the pulp chamber of the right mandibular first molar, largely removed the pulp of the distal root canal by using a #10-#15 K file within the working length, irrigated the distal root canal with PBS, left the chamber exposed to saliva from the oral cavity of rats for 10 min to introduce saliva into the root canal, dried the root canal with a paper point, delivered PBS within the apical part of the half working length, dried the pulp chamber and crown, and finally sealed them carefully with composite resin (SHOFU INC, Beautiful Flow Plus) (Supplementary Fig. 2b). Experimental samples were harvested 2 weeks, 4 weeks and 6 weeks after the endodontic procedures. The controls were healthy rat mandibles without endodontic procedures at the same age as the experimental samples. Samples with or without endodontic procedures at one time point were defined as one group.

### Rat sample harvest

Mandibles of rats were freshly harvested and fixed immediately in 4% paraformaldehyde at 4 ℃ for 2 days, decalcified using 10% EDTA and embedded in paraffin. The position of the sagittal section was located through the distal root (Supplementary Fig. 2a), and 5-µm thick sections were used for histological staining and immunohistochemical analysis. The framed area (Supplementary Fig. 2a3) is the magnified distal area of the µCT image, with histological and immunohistochemical staining in Figs. 3 and 5–7.

### µCT analysis

Rat samples were scanned by SCANCO MEDICAL µCT50 (70 KV, 114 µA, 8 w, 15 µm). A green frame of 600 μm × 600 µm adjacent to the apex was selected as the region of interest (ROI) of the apical area (Fig. 3a–c), and 20 serial layers forward and backwards were selected for analysis of BV/TV, Tb.Th, Tb.Sp. and Tb.N.^46^ Each group contained four individual samples.

### Histological and immunohistochemistry staining

Briefly, paraffin blocks were sectioned at a thickness of 5 µm. Serial sections were stained with haematoxylin (Biosharp, BL702A) and eosin (Beyotine, C0105-2) staining (H&E), TRAP staining (Sigma, 378 A) following the manufacturer’s instructions, Masson’s trichrome staining and immunohistochemical staining. Masson’s trichrome staining protocol from the IHC world was modified using Weigert’s iron haematoxylin solution, Ponean red, acid fuchsin (A121933), phosphomolybdic acid (KESHI 110900304), phosphotungstic acid (JINMAO 1148322) and Aniline blue (Huaxia Reagent 28631-66-5).

Immunohistochemistry was conducted using Vector (PK-6101 Elite) following the manufacturer’s instructions; briefly, 3% hydrogen peroxide (BOSTER, AR1108) for 10 min, blocking 10% goat serum, primary antibody rabbit anti-Osterix (Abcam, #22552)1:500, rabbit anti-pSmad2/3 (Santa, sc-11769-R), at 4 ℃ overnight, secondary antibody (1 h), ABC reagent (1 h), and DAB (ZLI-9018) development.

As reported in the literature,^47^ N.Osx^+^ cells/T.Ar (number of Osx-positive cells in the apical ligament/periodontitis area), N.Osx^+^ cells/B.Pm (number of Osx-positive cells on the bone surface), N.TRAP^+^ cells/B.Pm (number of TRAP-positive cells on the bone surface) and N.TRAP^+^ cells/T.Ar (number of TRAP-positive cells in the region of interest) were measured for statistical analysis. The ROI is designated by the boxed area in (Fig. 6a1–a4) and (Fig. 7a1–a4); three discontinuous sections were selected per sample, and three individual samples were included per group.

### Statistical analysis

Statistical significance was assessed using two-tailed Student’s *t*-test for comparisons between two groups. *P*-values less than 0.05 were considered significant (marked with *), and *P*-values less than 0.01 were considered significant (marked with **). Data are presented as the mean ± standard deviations (SDs) unless otherwise indicated.

## Supplementary information

Supplemental information

Supplemental figure 1

Supplemental figure 2
