# Supplementary material for: Disturbed bone remodelling activity varies in different stages of experimental, gradually progressive apical periodontitis in rats
Source: Int J Oral Sci. 2019 Aug 26;11(3):27. doi: 10.1038/s41368-019-0058-x (PMC6802676; doi:10.1038/s41368-019-0058-x)
Supplement: Supplementary file 1 — Supplemental information [file 41368_2019_58_MOESM1_ESM.doc]

**Supplementary information**

**Supplementary Figure 1: Aberrant activation of TGF-β signaling pathway.**

(**a**) Representative immunohistochemistry staining of pSmad2/3 of human apical region tissue from patients without (Control) or with chronical apical periodontitis. Red arrow indicates generally increased pSmad2/3 positive cells. (**b**) Representative immunohistochemistry staining of pSmad2/3 in distal apical region of rats’ mandibular first molar with or without induction of EAP two weeks of observation. Black dotted box in navigation is magnified on its right. pSmad2/3 positive cells were increased on bone surface (red arrow) and in blood vessels (blue arrow). Statistic analysis is located on the lower panel, N.pSmad2/3^+^ cells/T.Ar (number of pSmad2/3 positive cells in apical area), N. pSmad2/3^+^ cells/B.Pm (number of pSmad2/3 positive cells on bone surface). Blood vessels (blue asterisk); bone surface (grey dotted line); m, mesial; d, distal; f, furcation area; ap, apical bone; av, alveolar bone. Scale bar: 100µm.

**Supplementary Figure 2: Schematics.**

(**a**)Navigation of section. Sagittal view of the mandibular first molar of rats in X-ray film, transverse plane (**a1** red line), sagittal section (**a2** red line), framed box of distal apical area in sagittal section(**a3**) are representative images in **Fig3,5,6,7**. m, mesial; d, distal; c, crown; mr, mesial root; dr, distal root;br, buccal root; lr, lingual root; f, furcation area; ap, apical bone; av, alveolar bone. (**b**) Schematics of modified endodontic procedures of experimental apical periodontitis. (**a1**)Access to pulp chamber (**a2**) Removal of distal dental pulp (**a3**) Irrigation. (**a4**) Drying. (**a5**) Adding PBS. (**a6-8**) Sealing cavity. (**c**) Schematics of disturbed bone remodeling at different stages of experimental apical periodontitis. (**c1**) Homeostasis (bone marrow cells, purple). (**c2**) PDL is widened (red arrow), osteoblasts (brown) and osteoclasts (dark red) are increased, while adjacent bone marrow are decreased. (**c3**)Large bone lesion forms, increased osteoblasts are located off bone surface into apical lesion associated with fibers, while adjacent bone marrow are replace by increased osteoblasts and fibers (pink). Dark orange, necrotic pulp residual in root canal and accessary root canal.
