# Supplementary figures and images for: Disturbed bone remodelling activity varies in different stages of experimental, gradually progressive apical periodontitis in rats
Source: Int J Oral Sci. 2019 Aug 26;11(3):27. doi: 10.1038/s41368-019-0058-x (PMC6802676; doi:10.1038/s41368-019-0058-x)

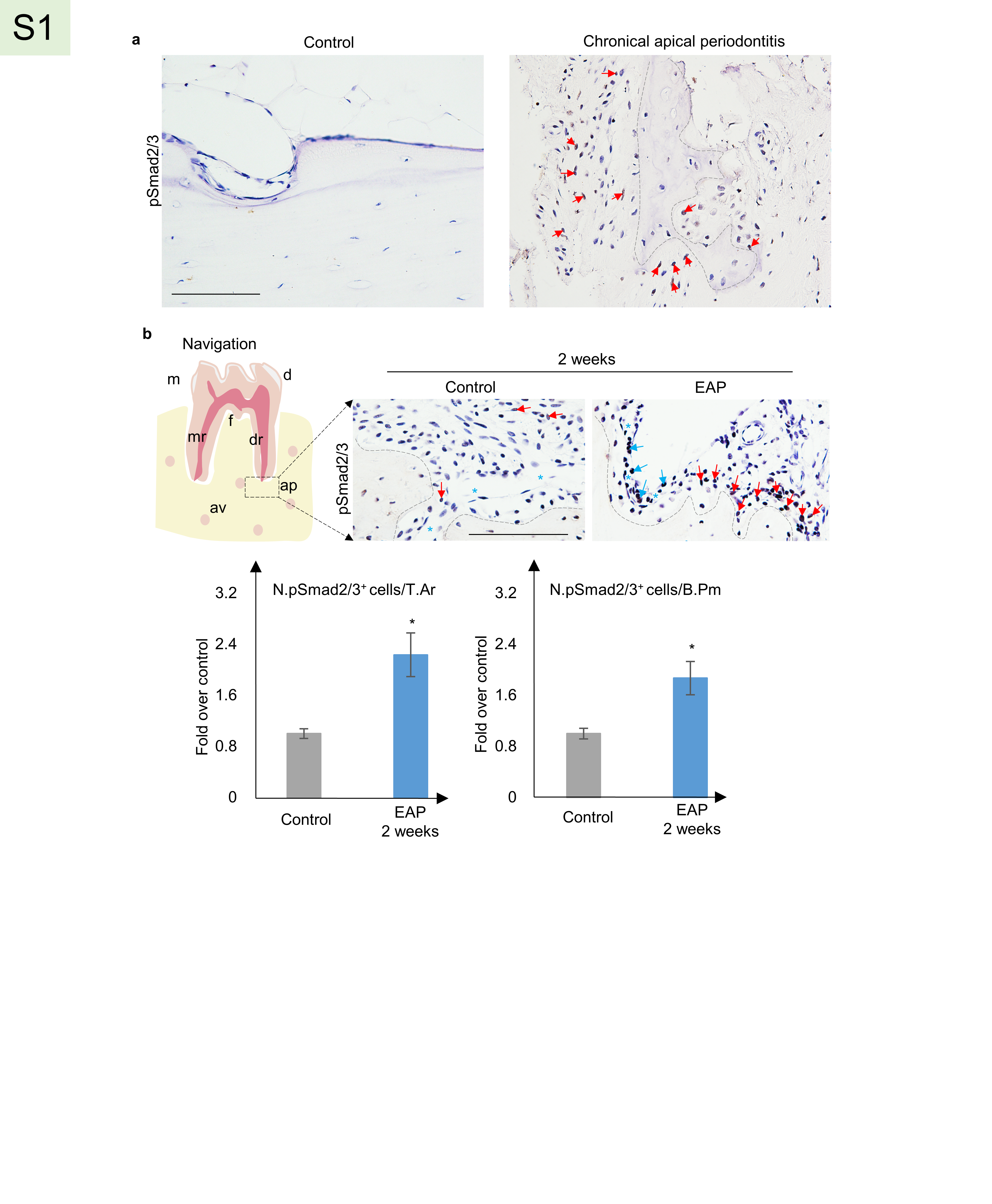

Supplement: Supplementary file 2 — Supplemental figure 1 [file 41368_2019_58_MOESM2_ESM.tif]

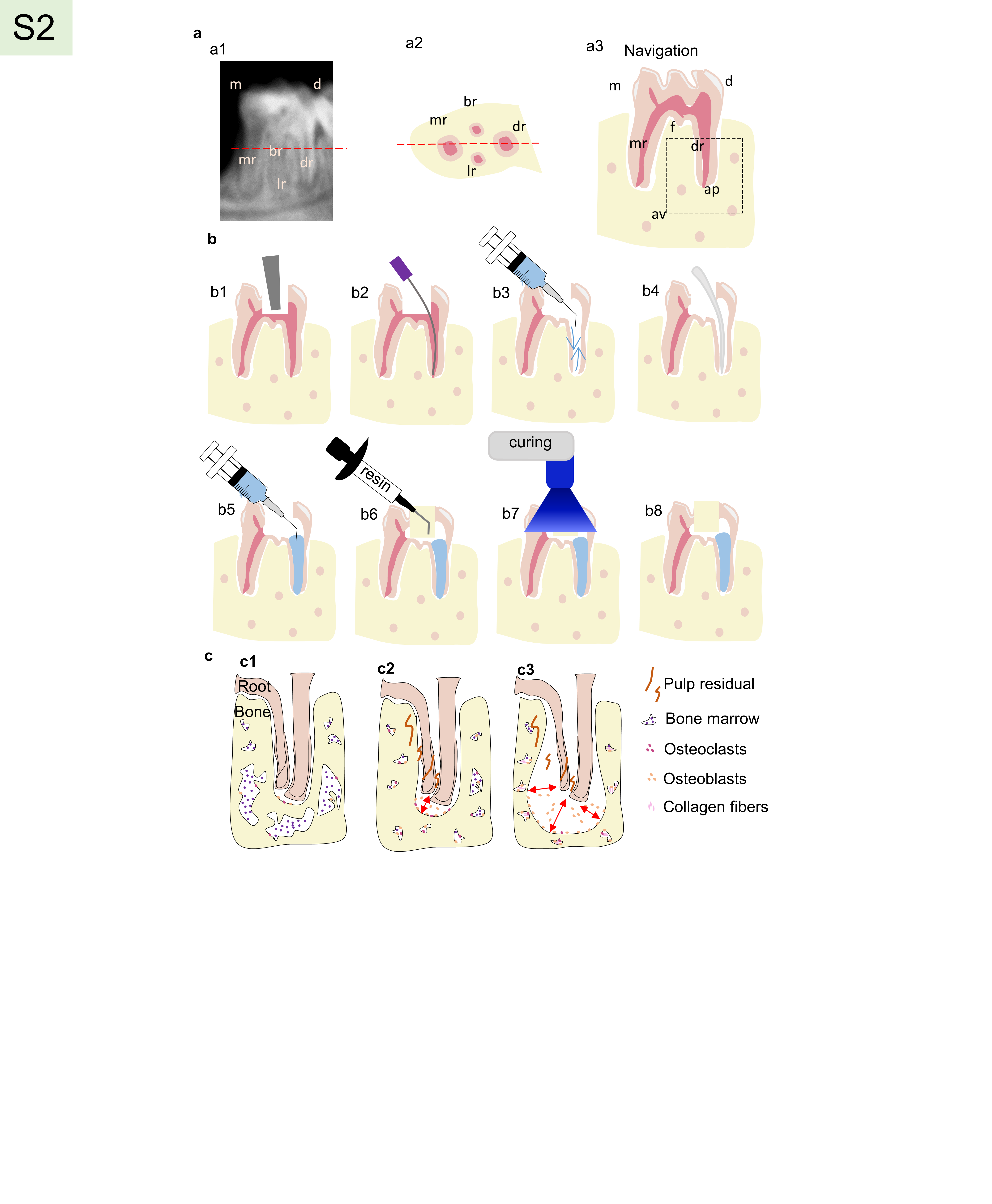

Supplement: Supplementary file 3 — Supplemental figure 2 [file 41368_2019_58_MOESM3_ESM.tif]
